# New Insights into the Pathogenesis of Non-Alcoholic Fatty Liver Disease: Gut-Derived Lipopolysaccharides and Oxidative Stress

**DOI:** 10.3390/nu12092762

**Published:** 2020-09-10

**Authors:** Domenico Ferro, Francesco Baratta, Daniele Pastori, Nicholas Cocomello, Alessandra Colantoni, Francesco Angelico, Maria Del Ben

**Affiliations:** 1 I Clinica Medica, Department of Clinical, Internal, Anesthetic and Cardiovascular Sciences, Sapienza University of Rome, 00185 Rome, Italy; domenico.ferro@uniroma1.it (D.F.); daniele.pastori@uniroma1.it (D.P.); nicholas.cocomello@gmail.com (N.C.); alessandracolantoni@libero.it (A.C.); maria.delben@uniroma1.it (M.D.B.); 2 Department of Public Health and Infectious Diseases, Sapienza University of Rome, 00185 Rome, Italy; francesco.angelico@uniroma1.it

**Keywords:** non-alcoholic fatty liver disease, lipopolysaccharide, oxidative stress, cardiovascular risk

## Abstract

Non-alcoholic fatty liver disease (NAFLD) is the most common chronic liver disease worldwide. The intricate NAFLD pathogenesis is summarized by the multiple-hits hypothesis, which combines all the environmental and genetic factors that promote the development of NAFLD into a single scenario. Among these, bacterial lipopolysaccharides (LPS) are derived from the overgrowth of Gram-negative bacteria and translocated mainly as a consequence of enhanced intestinal permeability. Furthermore, oxidative stress is increased in NAFLD as a consequence of reactive oxygen species (ROS) overproduction and a shortage of endogenous antioxidant molecules, and it is promoted by the interaction between LPS and the Toll-like receptor 4 system. Interestingly, oxidative stress, which has previously been described as being overexpressed in cardiovascular disease, could represent the link between LPS and the increased cardiovascular risk in NAFLD subjects. To date, the only effective strategy for the treatment of NAFLD and non-alcoholic steatohepatitis (NASH) is the loss of at least 5% body weight in overweight and/or obese subjects. However, the dose-dependent effects of multispecies probiotic supplementation on the serum LPS level and cardiometabolic profile in obese postmenopausal women were demonstrated. In addition, many antibiotics have regulatory effects on intestinal microbiota and were able to reduce serum aspartate aminotransferase (AST), alanine aminotransferase (ALT), and tumor necrosis factor alpha (TNF-α) in NASH animal models. Regarding the oxidant status, a Mediterranean diet has been reported to reduce oxidant stress, while vitamin E at high daily dosages induced the resolution of NASH in 36% of treated patients. Silymarin had the positive effect of reducing transaminase levels in NAFLD patients and long-term treatment may also decrease fibrosis and slow liver disease progression in NASH. Finally, the influence of nutraceuticals on gut microbiota and oxidant stress in NAFLD patients has not yet been well elucidated and there are insufficient data either to support or refuse their use in these subjects.

## 1. NAFLD: An Emerging Multifactorial Liver Disease

Non-alcoholic fatty liver disease (NAFLD) is the most common chronic liver disease in the world [1]. NAFLD encompasses a wide spectrum of liver diseases, from simple steatosis to non-alcoholic steatohepatitis (NASH) [2]. The reported prevalence of NAFLD is 25–30% in Western countries [1,3] and 12–24% in Asia [4]. Currently, in the United States, NASH is the leading cause of liver transplantation in women and is predicted to become the leading cause in males as well [5]. These findings underline the global importance of understanding the disease’s pathology.

At first, a “two-hit” hypothesis was proposed to describe NAFLD’s pathogenesis. The first hit results from insulin resistance, which causes liver steatosis with increased hepatic lipogenesis but impaired free fatty acid degradation. Fat accumulation sensitizes the liver to induce inflammation and cell death by a second pathogenic insult that promotes oxidative stress, where the latter also caused by changes in the gut microbiota [6,7], culminating in NASH and fibrosis [8,9]. More recently, an updated and widely recognized theory called the “multiple-hit hypothesis” has been proposed to summarize the intricacy of NAFLD’s pathogenesis and define all factors that act in parallel when causing the onset and progression of NAFLD [10]. Among the multiple parallel hits, oxidative stress is considered to be the main contributor to liver injury and disease progression in this setting [11,12]. Factors promoting oxidative stress in liver diseases may be exogenous—such as viruses, alcohol, and drugs—or endogenous—such as insulin resistance, obesity, and diabetes.

NAFLD, which is especially associated with advanced fibrosis, is strongly associated with a significantly increased risk of end-stage liver disease and hepatocellular carcinoma [13]. However, people with NAFLD have an increased chance of developing cardiovascular diseases, which represents the major causes of death in this setting [14]. A meta-analysis of 16 cohort studies with a median 7-year follow-up revealed that patients with NAFLD had a 64% additional risk compared with patients without NAFLD of having fatal or nonfatal cardiovascular events, such as a myocardial infarction, stroke, angina, or coronary revascularization [15]. Moreover, a systematic review and meta-analysis of 34 studies showed that NAFLD was associated with an increased risk for incident cardiovascular disease (CVD) (hazard ratio (HR) = 1.37), and specifically, an increased risk for coronary artery disease (HR = 2.31) and hypertension (HR = 1.16), but was not associated with CVD-related mortality and overall mortality when compared with patients without NAFLD [16]. Finally, several studies demonstrated a strong association between NAFLD and non-invasive surrogate markers of atherosclerosis, such as the carotid intima-media thickness, brachial artery flow-mediated dilation, and the coronary calcium score [17,18,19].

These findings fit with the hypothesis that NASH contributes to a higher risk of CVD as a component of the metabolic syndrome (MetS). However, whether the close association between NAFLD and CVD is explained by the atherogenic profile of MetS or as an independent involvement of NAFLD in the pathogenesis of cardiovascular disease is still debated [20,21].

## 2. NAFLD Pathogenesis

### 2.1. Gut Microbiota

Increasing evidence shows that the gut microbiome plays a significant role in human metabolism, health, and disease [22], suggesting that the gut microbiome could be considered a metabolic organ in the host.

In patients with NAFLD, the microbiome’s abundance [23] and composition are altered (dysbiosis) [24]. The crucial role of the gut microbiome in the NAFLD pathogenesis is highlighted by the fact that fecal transplantation from mice with NAFLD into wild-type mice caused NAFLD in the latter [25]. In addition, the dysbiosis of the microbiota changes with the disease severity [26].

The role of lipopolysaccharides (LPS) derived from Gram-negative bacteria surfaces has been widely investigated in liver diseases [27] and elevated serum levels of LPS have been demonstrated in NAFLD [28,29]. Patients with NAFLD showed an increase of 38–40% of their LPS serum level in comparison to dysmetabolic patients without NAFLD [30,31]. This increase in circulating LPS may be the consequence of several factors. The overgrowth of intestinal Gram-negative bacteria and an increased intestinal permeability has been found [23,32,33]. In particular, Miele and colleagues demonstrated a doubled intestinal permeability in patients with NAFLD and a tripled prevalence of small intestinal bacterial overgrowth (SIBO) in the same patients [34].

Dietary habits may explain the increased serum LPS in patients with NAFLD. Diets rich in fat may favor LPS translocation via different mechanisms. Unsaturated fatty acids have been reported to be able to modulate intestinal integrity, reducing the expression of tight junction proteins and altering their distribution, both directly and through the interaction with intestinal epithelial cells [35]. In another study, Awada et al. demonstrated that oxidized fatty acids induce the apoptosis of enterocytes, impairing the barrier integrity [36]. A further mechanism that may cause hyperpermeability in patients with NAFLD is the higher production of bile acids (BAs) induced by high-fat diets (HFDs) [37]. BAs, which stimulate the epidermal growth factor receptor (EGFR), lead to an impaired gut permeability [38]. Finally, several studies demonstrated that an HFD alters the abundance and quality of gut microbiota. However, the mechanism through which these alterations induce intestinal barrier alterations is still debated [35]. Overall, these mechanisms may partially explain the “leaky gut” phenomena observed in most patients with NAFLD [33].

Another LPS translocation method is represented by a mechanism of cotransport with chylomicrons [39]. Vors et al. [40] demonstrated that LPS absorption with chylomicrons may depend both on the lipid content of the diet and on the subject’s chylomicron clearance ability (indeed, the serum LPS peak after a high-fat meal was lower in lean versus obese men).

**Figure 1:**
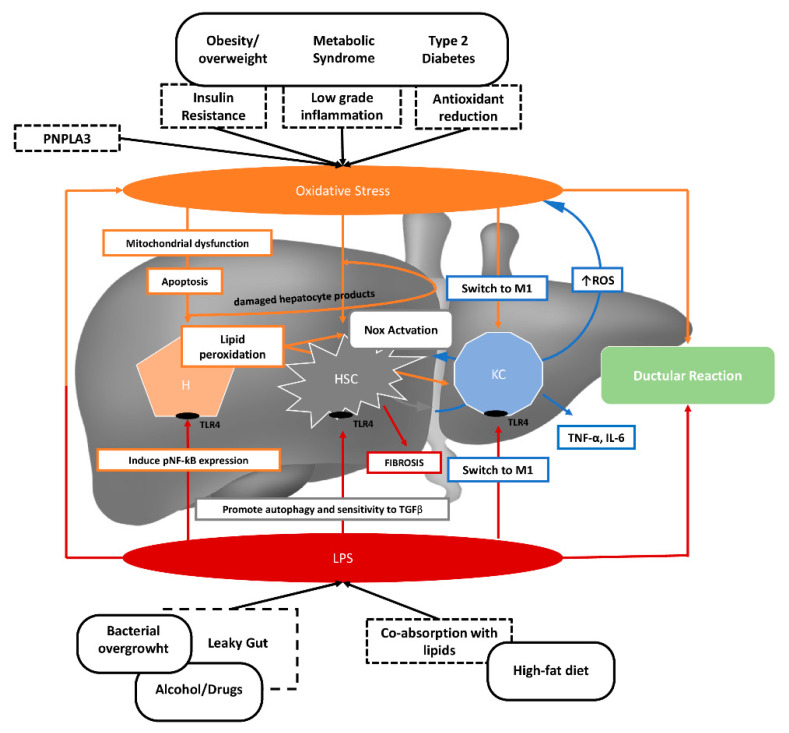
The role of increased serum lipopolysaccharides (LPS) and oxidative stress in the pathogenesis of non-alcoholic fatty liver disease. Dysmetabolic conditions, such as obesity, metabolic syndrome, and type 2 diabetes may induce oxidative stress as a consequence of increased insulin resistance, low-grade inflammation, and a reduction in antioxidants. Bacterial overgrowth, excessive alcohol consumption, and a diet rich in fat may induce increased intestinal LPS translocation. Oxidative stress and LPS promote hepatocellular damage, as well as hepatic stellate cell and Kupfer cell activation. PNPLA3: Patatin-like phospholipase domain-containing protein 3; Nox: NADPH oxidase; M1: M1 macrophages; ROS: Reactive oxygen species; HSC: Hepatic stellate cell; KC: Kupfer cell; TLR4: Toll-like receptor 4; pNF-κB: Phosphorylated nuclear factor κB; TNF-α: Tumor necrosis factor α; IL-6: interleukin 6; TGFβ: Transforming growth factor β.

This translocation increases the risk of NAFLD development through the activation of hepatic inflammatory cells [23,29,41]. Bacterial endotoxins are recognized by toll-like receptors (TLRs) on hepatocytes, hepatic stellate cells, and Kupfer cells [29]. When bacterial LPS signal through TLR4, the signaling ultimately activates the nuclear factor kappa-light-chain-enhancer of activated B cells (NF-κB) and the subsequent inflammasome activation [29]. In addition, endotoxins could directly damage hepatocytes and activate Kupffer cells to produce inflammatory cytokines, followed by the waterfall effect and release of oxygen radicals (Figure 1).

### 2.2. Oxidative Stress

Oxidative stress reflects the imbalance between the production of reactive oxygen species (ROS) and the scavenging capacity of the antioxidant system in favor of the former [42]. At high concentrations, ROS cause oxidative modifications to cellular macromolecules (DNA, lipids, proteins, etc.) and lead to the accumulation of damaged macromolecules, inducing liver injury [43]. Thus, the mechanism through which ROS contribute to NAFLD progression may be related to both indiscriminate oxidative biomolecular damage and dysregulated redox signaling [44], although the specific molecular pathways are not fully understood.

Oxidative stress may also have deleterious effects on antioxidant defense mechanisms. In fact, the overproduction of ROS may directly inhibit the activities of antioxidant enzymes, such as superoxide dismutase (SOD) and catalase (CAT), or deplete antioxidant molecules, such as glutathione (GSH) [45].

The hepatic detoxification process is the main source of oxidative stress in the liver. The liver has many functions; one of the most important is the ability to filter the blood and detoxify potential harmful eso- or endotoxins. Several substances, such as alcohol, toxins, drugs, and food, undergo the hepatic detoxification process [46]. Biotransformation reactions physiologically generate intermediate ROS to allow for the oxidation of toxins and facilitate their detoxification and excretion [47]. Therefore, under normal conditions, the amount of generated ROS is exactly what is needed for the detoxification process. Many antioxidant cofactors are also necessary to counterbalance the ROS production. By contrast, an overload of toxins or a shortage of endogenous antioxidant molecules may lead to oxidative stress, which in turn may induce tissue damage and promote inflammation. Oxidative stress increasingly appears as one of the most important pathological events during NAFLD development and the link between simple steatosis and NASH manifestation [48].

Oxidative stress is associated with many chronic diseases, especially those characterized by low-grade inflammation, such as diabetes, metabolic syndrome, and obesity. Oxidative stress is also an important factor for the pathogenesis and progression of cardiovascular disease, and it has been suggested that it may be a possible mechanism linking NAFLD to cardiovascular disease [48]. In fact, only a minority of NAFLD patients experience advanced liver disease, while cardiovascular events represent the leading cause of morbidity and death in this clinical setting.

NADPH oxidase (Nox) is considered to be the most important cellular source of oxidative stress, and its activation has been associated with the possible development of liver damage. The isoforms of NADPH oxidase, which include Nox1, Nox2, and Nox4, regulate the activation of hepatic stellate cells and hepatic apoptosis, which are two central aspects in the fibrogenic process [49].

Our group has assessed the oxidative stress in subjects with NAFLD. In a study first, we observed increased oxidative stress in vivo in a large cohort of subjects with NAFLD. This was documented by measuring the urinary levels of 8-iso-prostaglandin F2α (8-iso-PGF2α), which results from the non-enzymatic oxidation of arachidonic acid, and the serum levels of the soluble Nox2-derived peptide (sNox2-dp), which represents an indicator of Nox2 activation, the main isoform of NADPH responsible for ROS production. In this study, the increases in the urinary levels of 8-iso-PGF2α and the serum sNox2-dp levels observed in NAFLD were independent of the presence of obesity, diabetes, and metabolic syndrome, and increased progressively with the increasing severity of liver steatosis at ultrasound [50]. Furthermore, in the same series, the urinary levels of 8-iso-PGF2α were independently associated with the serum levels of cytokeratin-18, which is a validated liver marker of apoptosis [51]. More recently, on a wider series of subjects with NAFLD, we have shown a significant reduction in the plasma levels of vitamin E, both in subjects with simple steatosis and in those with NASH, suggesting the presence of increased oxidative stress, even in the earlier stages of the disease. Vitamin E is an important antioxidant fat-soluble vitamin, which protects cell membranes and lipoproteins from peroxidation. It has been hypothesized that the presence of increased oxidative stress in NAFLD may induce a reduction in natural antioxidants because of their excessive consumption [52].

## 3. Lipopolysaccharide and Oxidative Stress

**Figure 2:**
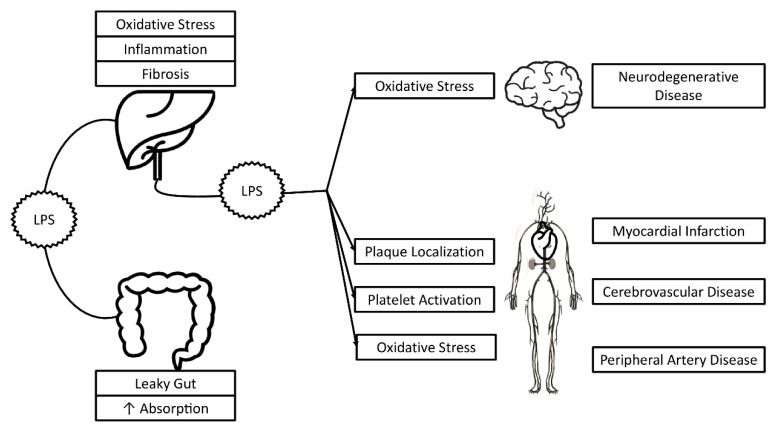
Possible effects of LPS in non-alcoholic fatty liver disease and non-hepatic disorders.

As was already mentioned, some data suggest that LPS helps to induce oxidative stress in different clinical settings, such as atherosclerosis [53] and neurodegenerative disease [54] (Figure 2).

In particular, our group showed in an in vitro study that an LPS concentration similar to that detected in atherosclerotic plaque resulted in a dose-dependent TLR4-mediated Nox2 up-regulation by human monocytes, increasing sNox2-dp by about 4.5-fold [53]. This concept was confirmed in several animal models, where alterations of gut microbiota influenced Nox2 activation and redox signaling [55,56,57]. Moreover, it was also recently reported that in patients at risk of cardiovascular events, the circulating LPS concentration independently predicted myocardial infarction, stroke, and cardiovascular death during a follow-up of about 3 years [58]. Finally, in subjects affected by neurodegenerative diseases, high Nox2 activation was shown and its correlation with LPS suggests a role of the gut microbiota as a source of oxidative stress [54]. We also demonstrated that gut-derived LPS increase post-prandial oxidative stress via Nox2 activation in patients with impaired fasting glucose, with an approximately 30% increase in serum LPS and a 36% increase in circulating markers of oxidative stress after a meal [59].

Several studies investigated the LPS role in liver modifications. Carpino et al. found an increased LPS localization into liver cells from biopsy-proven human and experimental NAFLD, which was significantly associated with liver inflammation through the TLR4 pathway [30]. The same pathway seems to be involved in LPS-induced macrophage and platelet activation. Moreover, in a previous study, we observed an association between systemic oxidative stress and some severity indices of fibrosis in liver biopsies of NAFLD patients [60]. In fact, we found a significant correlation between hepatic stellate cell niche activation and systemic oxidative stress markers (i.e., serum F2-isoprostanes and Nox2 activity). Finally, in a recent report, we showed that NAFLD patients in the highest sNox2-dp tertile, which is a marker of oxidative stress, had the highest LPS values (with an OR of 4.71 that was found using multivariate analysis) [31].

Altogether, these data suggest that increased LPS concentrations could induce enhanced lipid peroxidation through oxidative stress impairment. Therefore, we believe that oxidant stress could represent a link between LPS and increased cardiovascular risk in NAFLD subjects.

## 4. Therapeutic Approach to Reduce Oxidative Stress and Lipopolysaccharide in NAFLD

To date, the only effective strategy for the treatment of NAFLD and NASH is the loss of at least 5% of body weight in overweight and/or obese subjects, although many therapeutic approaches have been proposed. However, so far, no drugs have been approved for the treatment of NASH, no specific treatments can be recommended, and any drug treatment is off label [61].

### 4.1. Therapeutic Approach to Reducing Lipopolysaccharides in NAFLD

Few studies have been performed to assess whether LPS serum levels could be affected by dietary interventions.

Dietary patterns reflecting healthy food choices seem to be associated with lower serum LPS activity and a Mediterranean diet pattern, rich in unsaturated fats and fiber, has been suggested as a dietary strategy to reduce endotoxemia [62]. In fact, in a nutritional survey of 668 individuals with type 1 diabetes in the Finn Diane Study, healthy dietary choices, such as the consumption of fish, fresh vegetables, and fruits and berries, were associated with reduced systemic endotoxemia [63]. Moreover, placing eight healthy subjects on a Western-style diet for 1 month induced a 71% increase in plasma levels of endotoxin activity, whereas a prudent diet reduced levels by 31% [64]. In a further study, the consumption of a low-fat high-carbohydrate diet enriched in n-3 polyunsaturated fatty acids (PUFAs) for three weeks reduced the LPS fasting plasma (0.24 ± 0.01 EU/mL) levels when compared with a Mediterranean diet (0.38 ± 0.06 EU/mL) and a high saturated fat diet (0.35 ± 0.03 EU/mL) in twenty older adults [65].

Postprandial endotoxemia, after meals with different fatty acid compositions, has been evaluated in several studies. Overall, these studies show increased LPS serum levels after high saturated fat meals, suggesting the different effects of dietary fats on the regulation of the intestinal epithelial endotoxin transport [66] and the postprandial low-grade inflammation [67,68]. However, the link between dietary patterns, intestinal microbiota, sub-clinical inflammation, and endotoxemia is still debated, although most evidence shows a positive association with high-fat diets [69]

Regulating gut flora with probiotics or prebiotics has become a new approach used to prevent and treat several metabolic diseases, such as NAFLD. Probiotic bacteria are known to reduce pathogenic bacterial growth and restore the integrity of the intestinal barrier against LPS-induced epithelial toxicity [70].

Previous data showed that serum LPS, liver TLR4-mRNA, and serum inflammatory cytokines in a probiotics intervention group were all significantly decreased compared to that in the NAFLD model group. Additionally, the degree of liver steatosis and inflammatory cell infiltration in the intervention group was also reduced relative to the model group; based on these results, it was speculated that probiotics may delay the process of NAFLD by inhibiting the LPS–TLR4 signaling pathway [70]. In a narrative review, Eslamparast and colleagues summarized the studies that show that probiotic supplementation in animal models and human studies improve the inflammatory status and clinical manifestations in NAFLD [71]. Recently, the dose-dependent effects of multispecies probiotic supplementation on the serum LPS levels and cardiometabolic profile in obese postmenopausal women were demonstrated [72].

Many antibiotics have regulatory effects on intestinal microbiota and are of benefit to NAFLD [73]. For example, oral treatment with Cidomycin was found to promote the small intestine transit rate and reduce serum levels of alanine aminotransferase (ALT), aspartate aminotransferase (AST), and tumor necrosis factor alpha (TNF-α) in a NASH mouse model, indicating the potential of Cidomycin in alleviating the severity of NASH via intestinal microbiota modulation [74]. Rifaximin, which is a largely water-insoluble and nonabsorbable (<0.4%) drug, has been shown to exert antimicrobial activity against enteric bacteria, such as *Streptococcus*, *Bacteroides*, and *Citrobacter* [75]. Gangarapu et al. have demonstrated that a short-term administration of rifaximin (1200 mg/day for 28 days) improved the clinical status of patients with NAFLD/NASH, which was associated with reduced serum transaminases and circulating endotoxins [76]. Abdel-Razik et al. reported that after rifaximin therapy (1100 mg/day for 6 months), patients with NASH showed significantly reduced levels of proinflammatory cytokines, ALT, and NAFLD-liver fat score [77]. However, in an open-label clinical trial, rifaximin administration (800 mg/day for 6 weeks) was not effective for humans with NASH [78]. The inconsistency may be due to the small sample size, the relatively low treatment dose, or the short duration of the clinical study.

### 4.2. Therapeutic Approach to Reducing Oxidative Stress in NAFLD

Current evidence generally supports the benefits of a healthy diet on oxidative stress and chronic diseases. In fact, oxidative stress can be triggered by a high-level consumption of several macronutrients. Therefore, saturated fatty acids, omega-6 PUFA, and glucose may induce inflammation through nuclear factor NF-κB-mediated pathways [79]. Furthermore, diets rich in saturated fat, cholesterol, trans-fatty acids, and increased fructose can induce oxidative stress in NAFLD. In contrast, a protective role is played by the high consumption of plant-based foods (whole grains, cereals, seeds, nuts, legumes, vegetables, and fruits), and low consumption of meat, milk, and dairy products.

A Mediterranean diet (MD) is the best suggested dietary approach for the treatment of NAFLD according to guidelines [61]. Several dietary intervention trials have reported the favorable effects of MD on a variety of oxidative stress biomarkers reflecting different sources of oxidative stress (e.g., 8OHdG, malondialdehyde, and F2-iso-prostanes). Most studies showed reduced levels of oxidative stress in subjects treated with an MD [80,81,82,83,84,85]. In contrast, in a large, cross-sectional study of healthy women, healthy eating patterns were not associated with a lower level of oxidative stress, which was measured using fluorescent oxidation products as global markers of oxidative stress [86]. A large body of research has also investigated the potential beneficial effect of dietary antioxidants, such as olive oil, a greater ratio of omega-3/omega-6 PUFAs, polyphenols, carotenoids, and high-fiber foods, which may ameliorate oxidative stress in NAFLD [87,88,89].

The mechanism by which oxidative stress could be reduced following n-3 PUFA supplementation is still unresolved. The effect could be related to the immuno-modulatory and anti-inflammatory properties of n-3 PUFAs and to their ability to increase antioxidant enzymes, which could contribute to reducing the generation of ROS and other oxidative stress agents [90]. However, the differences in the treatment duration of randomized trials, doses, biomarkers for assessing oxidative stress make it difficult to form conclusions about the effectiveness of n-3 PUFAs at reducing oxidative stress. In a recent meta-analysis, data suggest that n-3 PUFA supplementation may be effective in the early stages of NAFLD, but not in patients with more severe NAFLD or NASH [91].

For patients with less severe NAFLD, several types of nutraceuticals have been suggested, mostly with antioxidant effects. In fact, anti-oxidative therapy using natural antioxidants represents a reasonable therapeutic approach for the prevention and treatment of liver diseases due to the role of oxidative stress in contributing to the initiation and progression of hepatic damage. The results of our previously reported studies showing increased systemic oxidative stress [50] and decreased vitamin E serum levels [52] in subjects with NAFLD suggest that early antioxidant treatment is beneficial, even in patients with simple fatty liver disease.

Vitamin E appears to be effective for the treatment of nondiabetic subjects with advanced NASH. In fact, in the “PIoglitazone versus Vitamin E versus placebo for the treatment of non-diabetic patients with Nonalcoholic Steatohepatitis (PIVENS) study”, vitamin E (800 IU/day) improved steatosis, inflammation, and ballooning and induced the resolution of NASH in 36% of patients (21% in the placebo arm) [92]. However, the high suggested daily dosages are a matter of concern due to the results of different metanalyses showing increased all-cause mortality and increased relative risks for hemorrhagic stroke [93] and prostate cancer [94] in those treated with high vitamin E dosages (higher than 400 IU/day). The PIVENS findings were not confirmed in 58 children and adolescents where vitamin E, supplied at 800 IU/day for 96 weeks, was not superior to a placebo, neither in attaining a sustained reduction in ALT levels or improving the liver histology [95]. Furthermore, Nobili et al. found no benefit due to vitamin E supplementation in a cohort of 53 children and adolescents with NAFLD [96].

Several other therapeutic approaches have been proposed to reduce oxidative stress. There are insufficient data to support the use of vitamin C in NAFLD patients, although vitamin C intake was reported to be inversely associated with the severity of NAFLD [97]. Moreover, polyphenols may present hepatoprotective effects because they reduce liver fat accumulation by decreasing oxidative stress, insulin resistance, and inflammation, and increasing fatty acid oxidation [98]. Silymarin is a valuable treatment option for liver diseases triggered by oxidative stress, such as alcoholic and non-alcoholic fatty liver disease and drug-induced liver disease. Silymarin may improve liver enzymes in the early stages of NAFLD. In a meta-analysis of 8 randomized clinical trials including 587 patients, Silymarin showed positive efficacy toward reducing transaminases levels in NAFLD patients [99]. Long-term treatment may also decrease fibrosis and slow liver disease progression in patients with NASH [100].

Thus, the influence of nutraceuticals on gut microbiota and oxidative stress in NAFLD patients has not yet been elucidated and there are insufficient data either to support or refuse the use of nutraceuticals to treat subjects with NAFLD.

## 5. Conclusions

Increased oxidative stress and gut-derived LPS blood levels have emerged as important factors in the pathogenesis of NAFLD and its progression to NASH. All these factors play a major role in proinflammatory molecule release and lipid peroxidation, leading to mitochondrial damage. LPS, the active component of endotoxin, is transported in the blood by chylomicrons and induced by a high-fat diet. However, little is known about factors inducing intestinal barrier alterations, gut bacterial translocation, or the subsequent hepatic TLR4 activation in NAFLD. Moreover, it is not yet clear which factors modulate variations in the intestinal permeability and LPS translocation during the progression to NASH.

As the consequence of ROS overproduction and a shortage of endogenous antioxidant molecules, high oxidative stress is a well-established cause of liver injury due to indiscriminate oxidative biomolecular damage and dysregulated redox signaling. NADPH oxidase may promote hepatic stellate cell activation in NASH, resulting in progressive fibrosis.

Several lines of evidence appear to suggest that LPS may induce oxidative stress activation in patients with NAFLD. Experimental and human studies have shown that alterations of gut microbiota may influence Nox2 activation and redox signaling. Moreover, a positive correlation between LPS blood levels and Nox2 levels in subjects with NAFLD has also been demonstrated.

Further randomized clinical trials could be useful to demonstrate a clear cause–effect relationship between LPS and oxidative stress in NAFLD.
